# Exploring the Attitudes of Medical Students Towards Social Media and E-professionalism in Al-Ahsa, Saudi Arabia

**DOI:** 10.7759/cureus.48718

**Published:** 2023-11-13

**Authors:** Saba Firdos, Sarah Almulla, Sarah Aldossary, Sarra Al Hassan, Latifah Aldhaif

**Affiliations:** 1 Clinical Neurosciences, King Faisal University, Al-Ahsa, SAU; 2 Medicine, College of Medicine, King Faisal University, Al-Hofuf, SAU

**Keywords:** medical students, e-professionalism, accountability, perception, intentions, social media

## Abstract

Background

In the digital age, social media is essential to everyone's life, including medical students. The rapid proliferation and updates in social media make this platform more attractive and accessible. However, online behavior and guidelines have always been a topic of ongoing debate. Thus, the study's objectives were to investigate the usage of social networking sites for various intentions among medical students, their opinions about applying these platforms for educational benefits, and their perception and accountability for e-professionalism.

Methodology

A cross-sectional study was conducted with the help of an online questionnaire among students of the College of Medicine at King Faisal University, Al-Ahsa, Saudi Arabia, from April to September 2023. Using a convenience sampling method, 577 students were invited to participate, with 97.1% (n = 560) agreeing to take part in the study. Both male and female students from all years, aged between 20 and 24 years, were included in this study.

Results

Nearly all medical students (99.5%, n = 557) were daily social media users. WhatsApp, YouTube, and Twitter (now X) were the most popular social media platforms, while Facebook was the least used. Gender differences were observed in platform preferences, with females favoring Instagram and Telegram and males preferring Reddit and Discord. Both genders utilize social media for various motives, including enjoyment (78.2%, n = 438), communication (68.4%, n = 383), knowledge (59.6%, n = 334), relaxation (43.6%, n = 244), and e-professionalism (12.5%, n = 70). Remarkably, 89% (n = 500) believed that social media could be a reliable medium for educational activities. Concerning e-professionalism, most students had private social media accounts, and a significant number believed their profiles did not portray them as professionals. However, a majority interacted with faculty members via social media. Male students exhibited a higher sense of e-professionalism responsibility, with many believing that online profiles should be considered during hiring decisions. Besides, female students believed online profiles could influence others' opinions about them.

Conclusion

Medical students are significantly involved with social media for different reasons. They acknowledge social media's potential benefits and enriching learning experiences. Nevertheless, a student's online behavior and attitude must be cognizant of and allied to the concept of e-professionalism, as it may directly lead to their future growth in medicine.

## Introduction

The term 'social media' refers to a group of internet-based applications (apps) that allow the creation and exchange of user-generated content. As technological advancements expand globally, social media has become a vital aspect of billions of lives worldwide, influencing nearly every industry [1]. Social media platforms have enabled people to communicate, connect, create, and share information without demanding high levels of coding expertise [2]. Based on the functions of social media, we can categorize them into collaborative projects like Wikipedia, blogs or microblogs such as Twitter (now X) and Tumblr, content communities like YouTube and Instagram, other social networking platforms like Facebook and LinkedIn, and specific virtual games with embedded social networking features [3, 4].

Social media has also been a valuable tool for academic purposes, such as learning, research, and staying up-to-date on current events. According to a study in Massachusetts, nearly all college and university students use social media regularly. Specifically, 98% have a Facebook account, and 84% use a school Twitter account. Two-thirds (66%) run a blog, while 41% offer a podcast. Nearly half (47%) of admissions staff take advantage of LinkedIn. Only 8% of people still use MySpace, but 85% use YouTube for their online presence [5]. A systematic review found that using social media platforms could lead to improved academic achievement, the development of positive professional attitudes, increased learning engagement, and more vital collaboration between peers and medical professionals [6]. As social media use continues to grow globally among the general population, healthcare professionals (HCPs) are likewise adopting these platforms [7]. The growing integration of social media in healthcare is supported by well-marked studies, for instance, in health and medicine [8]. There are a couple of studies examining the role of social media in bridging the communication gap between patients and HCPs or in enhancing interactions among HCPs [9-11]. Irrespective of educational background, clinical status, or place of practice, healthcare providers can connect through social media. This provides the opportunity for individuals to exchange their knowledge and experiences and the chance to cooperate in solving rare and unusual cases [12].

In contrast, social media also presents the risk of blurring personal and professional boundaries [13], as some students find it difficult to draw a clear line between personal and professional online interactions [13-15]. Therefore, in professional fields, students and practitioners are now navigating the newly emerged concept of e-professionalism, which pertains to the professional attitudes and behaviors they show in their online personas [16]. The concept of 'e-professionalism' was introduced as "attitudes and behaviors (some of which may occur in private settings) reflecting traditional professionalism paradigms that are manifested through digital media" by Cain and Romanelli [17]. The intersection between medical professionalism and social media has also been termed online professionalism or digital professionalism [18]. Indeed, developing professionalism during medical school sets the groundwork for a prosperous profession as a physician and ensures patients receive the highest quality care [19].

So far, numerous publications have revealed the unethical online conduct of medical students, educators, and HCPs [20]. For instance, extensive disclosure of one's self on social media platforms by HCPs is viewed as a flagrant breach of professional conduct [21]. Some studies show that HCPs may post inappropriate material on social media, including profanity, nasty remarks about coworkers, and patient information, and this behavior may be interpreted differently depending on the culture and setting [22]. Numerous studies have discovered instances of medical students posting pictures of drunkenness and drug use online [23]. Apparently, such posts violate patient privacy [24]. In some instances, students post rude remarks about coworkers and employers on different social media channels [25,26].

The rapid development of social media and the unexpected growth of its users have raised concerns about how medical students behave online and to what extent they are aware of the ethical standards that apply to social media use. Several strategies were recommended to facilitate the practice of e-professionalism, such as developing policies, creating training materials, monitoring online behavior, and staying up-to-date on the latest technologies [27]. The American Medical Association (2019) has also issued recommendations: physicians must uphold patient confidentiality online and refrain from posting any identifiable information. It's imperative to use social media ethically, distinguish between personal and professional content, and recognize the lasting nature of online posts. Online interactions with patients should always adhere to professional boundaries. Should physicians come across unprofessional content from colleagues, they must address or escalate the issue. Actions taken online can significantly influence a physician's reputation and the overall public's trust in the medical field [28]. Similarly, the Australian and New Zealand Medical Associations (2018) have developed guidelines to address the concerns of their students about social media usage, accountability, and professionalism [29].

Assuming the scarcity of published literature on this topic, research is needed to better understand and fill the potential gaps in the existing literature about the e-professionalism practices of medical students on social media. This study's objectives were to assess the usage of various social networking sites for different intentions and to investigate medical students' opinions towards using different social networking sites for educational purposes. Last but not least, to explore medical students' perceptions and accountability about e-professionalism.

## Materials and methods

Research design and participants

The present study conducted between April and September 2023, employed a cross-sectional research design among medical students from the College of Medicine at King Faisal University in Al-Ahsa, Saudi Arabia. Based on a 5% margin of error, a 5% significance level (type 1 error), and a 95% confidence interval, the required number of study participants was 577. The participants comprised both male and female medical students from all academic years. However, 17 students declined to participate. Of the 560 participants, 297 were female and 263 were male, with an average age of 20 to 24 years. The current study applied a convenience sampling method to select the participants.

Measures

Demographics and Details on Social Media Networking

A sheet was prepared to gather personal information such as age, gender, academic year, marital status, and nationality. We developed a structured questionnaire to measure the usage of social networking sites, including the frequency, types, and intentions of use. The questionnaire was designed based on research questions and study-based hypotheses such as: Do you use social media daily (e.g., Facebook, WhatsApp, Twitter, Instagram, Snapchat, YouTube, and others)? How many hours do you spend on social media? The researchers followed a systematic process to develop the questionnaire with the help of subject experts and peers. A total of five questions were designed to be asked of the proposed participants.

Perceptions Towards Accountability and E-professionalism on Social Media

The self-reported questionnaire was developed with the help of three questionnaires [30-32]. Seven questions were employed on students' perspectives on the use of social media in education, and seven were intended to investigate students' perspectives on social media use and privacy. Likewise, nine questions were adapted to study students' perspectives on accountability and e-professionalism. Overall, Cronbach's alpha reliability coefficient for the total score was 0.92, and the reliability coefficients for the domains ranged between 0.81 and 0.85.

Inclusion and Exclusion Criteria

Medical students from all academic years who were familiar with social media and actively engaged in the digital world for several reasons and understand the concept of e-professionalism were enrolled in the study. Non-medical students and medical students who are non-users of social media, and those who refused to participate, were excluded from the study.

Procedure

The researchers obtained ethical permission from the Research Ethics Committee, Deanship of Scientific Research, King Faisal University (approval no. KFU-REC-2023-MAR-ETHICS677). An electronic self-reported questionnaire was distributed via email and social networking accounts to the potential participants. Verbal consent was received from all medical participants before administering the questionnaire. The participants' privacy was protected by excluding identifying information from the questionnaire, such as students' names. Further, the collected data was stored in a password-protected file, and access to the file was restricted to the researchers only.

Data analyses

The current study employed SPSS Statistics version 26 (IBM Corp., Armonk, NY, USA) for the data analysis. All data underwent preliminary vetting to identify any missing values to ensure its quality and reliability. Descriptive statistics were used to summarize the participants' demographic details, with frequencies and percentages showing categorical variables and means and standard deviations for continuous ones. This study computed central tendency measures (mean, median) to estimate medical students' attitudes toward social media and e-professionalism. A chi-square test was employed to assess the association between categorical demographic factors (e.g., year of study, gender) and specific attitudes or behaviors on social media and e-professionalism.

## Results

**Figure 1:**
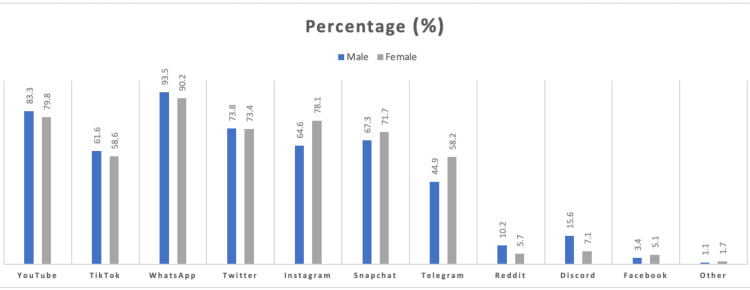
Frequency of using social media sites among male and female students

A total of 97.1% (n = 560) of medical students agreed to participate in the study. Of these, 51.5% (n = 297) were females, and 45.6% (n = 263) were males. Furthermore, 4.3% (n = 25) of the students were in their preparatory year, 61.5% (n = 355) of students were in their first three years, and 31.2% (n = 180) of students were in their final three years. Almost all (99.5%, n = 557) students were daily social media users. The majority (78%, n = 452) of the participants were between 20 and 24 years old and were single (91%, n = 522). Table 1 shows the baseline characteristics of participants in the study. Most students spend five to six hours on social media with no significant difference between males and females (females = 111 and males = 84), with a smaller number of students spending less than an hour (females = 3 and males = 7). As shown in Figure 1, the most widely used application among both groups was WhatsApp (91.7%, n = 514) followed by YouTube (81.4%, n = 456) and Twitter (73.5%, n = 412); Facebook was the least used social media application (4.2%, n = 24). For 78% (n = 231) of female and 65% (n = 170) of male students, there was a statistically significant correlation between female sex and the preference for Instagram and Telegram (p < 0.001). Contrarily, male students used Reddit and Discord much more than female students did, with 15.6% (n = 41) of male students using Discord and 10.% (n = 27) of male students using Reddit (p = 0.001 and p = 0.046, respectively).

**Table 1:** Baseline characteristics

Students per academic year (total n = 560)	Respondents			
	Male (n = 263)		Female (n =297)	
	n	%	n	%
Preparatory year (n = 25)	16	6.1	9	3
First year (n = 107)	56	21.3	51	17.2
Second year (n = 134)	46	17.5	88	29.6
Third year (n = 114)	38	14.4	76	25.6
Fourth-year (n = 65)	26	9.9	39	13.1
Fifth year (n = 90)	67	25.5	23	7.7
Intern (n = 25)	14	5.3	11	3.7

Table 2 shows that both categories of students utilize social media equally, with 78.2% (n = 438) using it for enjoyment, 68.4% (n = 383) for communication with family and friends, 59.6% (n = 334) for knowledge and education, 43.6% (n = 244) for relaxation, and 12.5% (n = 70) for e-professionalism. However, the male gender appears to be associated with using social media to make new friends (p = 0.002) and pass the time (p = 0.001). In contrast, the female gender exhibits a much greater rate of utilizing social media for knowledge and educational purposes (p = 0.041).

**Table 2:** Students' intentions for using social media platforms *p < 0.05 is statistically significant

Intentions	Male students		Female students		χ2	p
	n	%	n	%		
To be in touch with family, friends, and relatives	180	68.4	203	68.4	0.001	0.982
To pass the time	171	65	153	51.5	10.432	0.001
For fun	208	79.1	230	77.4	0.222	0.638
Make new friends	62	23.6	40	13.5	9.563	0.002
Knowledge and education	145	55.1	189	63.3	4.190	0.041
For relaxation (avoiding stress)	125	47.5	119	40.1	3.158	0.076
For e-professionalism (advertising myself)	31	11.8	39	13	0.230	0.631

The data in Table 3 indicates that there was no significant association between gender and students' perceptions of the use of social media in education. On the other hand, more than half of the students (89%, n = 500) thought that social media might potentially be a reliable medium for educational activities and enhance learning experiences (91%, n = 509).

**Table 3:** Students' perspectives on the use of social media in education *p <0.05 is statistically significant

Perspectives	Male students		Female students		χ2	p
	n	%	n	%		
Social media can be effectively used in classrooms to improve education	224	85.2	247	83.2	0.420	0.517
Social media is not useful for educational purposes in the curriculum	84	31.9	79	26.6	1.927	0.165
Social media has the potential to become an established channel for educational activities	233	88.6	267	89.9	0.249	0.618
Social media plays a role in preparing students for a career upon graduation	223	84.8	254	85.5	0.059	0.808
Social media needs to be incorporated more into coursework	181	68.8	213	71.7	0.561	0.454
Social media can improve learning experiences	239	90.9	270	90.9	0.000	0.989
Social media takes too much time to use for educational purposes	120	45.6	144	48.5	0.457	0.499

Table 4 displays the students' perceptions of privacy and social media use. More than half (78%, n = 439) of the students had their social media accounts set to private, with substantially more females (p = 0.043) than males doing so. This indicates that students were aware of the online privacy settings for social media. Additionally, both sexes agreed that their social media profiles do not appropriately portray them as professionals (58%, n = 322), while men were more likely to agree (p = 0.024). Students claimed they had shared any material on their social media pages that they did not want potential employers or faculty members to see (40%, n = 228, and 41.8%, n = 234, respectively). Additionally, most students (42.5%, n = 238) concurred that they shared some information on their social media profiles that they did not want their patients to see. However, a large proportion (72.3%, n = 405) were discovered to interact with faculty members via social media accounts.

**Table 4:** Students' perspectives on social media use and privacy *p < 0.05 is statistically significant

Perspectives	Males students		Females students		χ2	p
	n	%	n	%		
I use privacy settings to limit accessibility to my profile	216	82.1	223	75.1	4.087	0.043
My Twitter (or any social media) profile is an accurate presentation of me as a person	152	57.8	156	52.5	1.565	0.211
My Twitter (or any social media) profile is an accurate presentation of me as a professional	125	47.5	113	38	5.131	0.024
I have provided 'information' on Twitter (or any social media account) that I do not want a potential employer to view	102	38.8	126	42.4	0.766	0.381
I have provided 'information' on Twitter (or any social media account) that I do not want a faculty member to view	108	41.1	126	42.4	0.106	0.745
I have provided 'information' on Twitter (or any social media account) that I do not want a patient to view	112	42.6	126	42.4	0.001	0.969
I can communicate with a faculty member through social media	190	72.2	215	72.4	0.028	0.866

Table 5 illustrates students' perceptions of accountability and e-professionalism. More than half agreed that healthcare students should be more careful about their image online (86%, n = 480) and edit their online profiles before applying for a job (72%, n = 401). Most respondents felt that online profiles could affect people's opinions of them in both groups (84%, n = 471). Similarly, both groups agreed that they prefer not to share any information that affects their patients' view of them as future professionals (41%, n = 229). Males were found to be significantly different from females in that they were found to be more responsible and conscious of the idea of e-professionalism. They agreed that students should be held accountable for illegal acts (p = 0.011) and unprofessional behavior (p = 0.003). In addition, interestingly, they feel that their image on Twitter and other social media sites accurately presented them as future professionals (p > 0.001) and as individuals (p = 0.018), while most of the females believed otherwise. Furthermore, 60.8% (n = 160) of males thought Twitter or social media profile information should be considered in hiring decisions (p > 0.001). In contrast, 45% (n = 134) of females thought online profile information should not be used for hiring decisions. Similarly, females significantly (p = 0.042) believe that online profiles could affect people's opinions about them.

**Table 5:** Students' perspectives on accountability and e-professionalism *p < 0.05 is statistically significant

Perspectives	Males		Females		χ2	p
	n	%	n	%		
Should a student be accountable for an illegal act discovered through Twitter or (Social Media)?	193	73.4	188	63.3	6.523	0.011
Should a student be accountable for unprofessional behavior discovered through Twitter or (Social Media) postings?	176	66.9	162	54.5	8.927	0.003
If an employer of medical graduates chooses to review a prospective employee's Twitter or (Social Media sites), should the profile information be considered when making a hiring decision?	160	60.8	134	45.1	13.820	0.001
Does the image you present online through Twitter or (social media Sites) accurately present who you are as a person?	153	58.2	143	48.1	5.628	0.018
Does the image you present online through Twitter or (social media sites) accurately show who you are as a future professional?	148	56.8	113	38	18.621	0.001
Have you ever provided any 'information' (such as a photo, message, etc.) on Twitter or (social media sites) that you would not want a patient to view?	116	44.1	113	38	2.119	0.146
Do you think it is important to edit your social media profile before applying for a job?	186	70.7	215	72.4	0.191	0.662
Should healthcare students be more careful with their image online?	225	85.5	255	85.9	0.011	0.917
Do you think your online profile could affect people's opinion of you?	230	87.5	241	81.1	4.152	0.042

## Discussion

Social media is no longer just a tool for connecting with friends, family, and entertainment. We assumed that medical students and other professionals use it for various purposes and are aware of e-professionalism. Thus, the current study focused on three main issues, such as assessing the use of social media among medical students for different intentions, examining medical students' opinions towards using different social networking sites in education, and exploring medical students' perceptions of e-professionalism. The results show that 99.5% (n = 557) of participants were regular social media users, with five to six hours of engagement daily, with no significant difference.

Further, results revealed that a higher percentage of female students (78%, n = 231) preferred using Instagram, while a relatively lower number of male students (65%, n = 170) showed the same preference. This statistically significant correlation (p > 0.001) between females and their preference for Instagram shows evident gender differences in social media preferences. Moreover, the study also found that male students were more likely to use Reddit and Discord compared to female students. Men and women are known to exhibit subtle differences in social media usage, such as the amount of time spent on social media websites, the use of language, the reasons for joining social media platforms, and the nature and expression of emotions [33]. Numerous studies have examined students' social media usage patterns and purposes, focusing on gender differences. The current study found (Table 2) that 78.2% (n = 438) of students use social media for enjoyment, indicating that it is a source of entertainment for them. Another significant finding from the study was that 68.4% (n = 383) of students use social media to communicate with family and friends, highlighting its role in maintaining social connections and relationships. Moreover, the study revealed that 59.6% (n = 334) of students use social media for knowledge and education, suggesting that it is not just a platform for leisure activities but also for learning and acquiring information.

The study also found that there are significant gender differences in social media usage among students. Specifically, males are more likely to use social media to make new friends and pass the time. At the same time, females have a higher rate of utilizing social media for knowledge and educational purposes. Reversely, one study on university students' information-sharing behavior and social media use found that males are more likely to share learning resources and use social media for educational purposes more frequently than their female counterparts [34]. However, our study found that females are more likely to use social media for academic purposes than males. This could be due to several factors, such as a greater interest in learning, a stronger desire to succeed in school, or a supportive social network. Similarly, another study [35] found differences between male and female users regarding reliability, performance, awareness, and intention related to social media usage for learning purposes. The same results show that perceived usefulness and ease of use significantly predicted social media adoption. Still, there was no significant difference between males and females regarding social media adoption [36].

Significant differences were also found (Table 4) between both genders' perceptions of privacy settings for social media; female students revealed more concerns toward privacy. Supported research reports that a majority of medical students have their social media accounts set to private [37]. In addition, male and female students agreed that their social media profiles do not appropriately portray them as professionals [38]; significantly, male medical students agreed compared to female students. As reported, students are concerned about personal control and desire recommendations for appropriate content [39].

The present study shows (Table 5) that the majority of both male and female medical students acknowledged that students should be held accountable for illegal and unprofessional acts they post online on social media. In contrast, a study conducted in the United Arab Emirates showed that less than half of the participating pharmacy students (42.4%, n = 138) believed they were responsible for any unethical behavior displayed on social media profiles [32]. Interestingly, in one study, most pharmacy students agreed that students should be held accountable for the unethical and unprofessional behavior seen on social networking sites [40]. However, it is worth mentioning that in this study, males were significantly more aware and conscious of e-professionalism than their female counterparts. This may be because males are likelier to use digital communication platforms, including Twitter, than females.

Likewise, in our study (Table 5), males show a significant difference in image presentation through Twitter or social media as a professional (p > 0.001) and an individual (p = 0.018) compared to females. The study found that male Twitter users had more followers, tweeted more often, and had more retweets and mentions than females [41]. Regarding hiring decisions, male students considered social media and other online platforms, such as Twitter, as suitable recruitment platforms. Job seekers, as well as recruiters, are now commonly using social media. Companies are using social networks not only to market themselves but also as a tool in their recruiting, screening, and selection processes. Due to the unlimited reach of social media, students can consider social media as a recruitment platform. A study shows that students and employers recognize social media as a valuable tool in job search and recruitment but express reservations about data accuracy [42]. However, at the time, female students significantly (p = 0.042) believed that online profiles could affect people's opinions of them. This significant difference may be due to the gender and socio-cultural background of the female participants. This finding is supported by a study that reveals that female students put a lot of effort into organizing their Instagram profiles, carefully selecting images, and gaining recognition through likes and comments [43]. Conversely, research has shown that social media platforms are critical in hiring decisions. Jobs and even candidate rejection can adversely affect a person's online personality on social networking sites. Therefore, it's essential that hiring experts regularly scrutinize candidates' profiles on both professional networks such as LinkedIn, and personal platforms, including Facebook and Instagram [44].

Limitations

This study acknowledges several limitations. First, the selection of the sample was limited as the data was collected from a single college and city, which is insufficient for a comprehensive view. The expanded nature of the study might lead to different outcomes, and therefore, the results cannot be generalized. Also, a limited sample size could cause selection bias. A multidisciplinary approach would provide a high scope for comparative analysis between institutes and students from different specialties.

## Conclusions

Both genders believed in the potential of social media as a valuable educational tool for students; however, usage drives for social media were varied. Most students favored privacy concerns, particularly females, who are more likely to have private accounts. Concerns about online representation and e-professionalism were prevalent among medical students. Male medical students predominantly demonstrated better sensitivity to online accountability on platforms like Twitter and acknowledged that online profiles influence hiring decisions. Female medical students were also more apprehensive about how online profiles might shape public perceptions.

In light of these findings, it is recommended that workshops, seminars, and short courses be introduced to enhance career prospects through the strategic use of e-professionalism. It is also essential for educational bodies and institutions to formulate and implement comprehensive guidelines and policies. These should be intelligently designed to reflect social and cultural considerations, ensuring appropriate and effective engagement on digital platforms. Further, the study highlights several potential future research areas. One essential area is integrating e-professionalism training into the medical education curriculum. Besides, the impact of social and cultural factors on e-professionalism should be investigated due to its important implications.
